# Computational predictive processing models of consciousness: a systematic review of non-invasive brain signal analysis in disorders of consciousness

**DOI:** 10.3389/fncom.2026.1797090

**Published:** 2026-05-08

**Authors:** Sophie Adama, Martin Bogdan

**Affiliations:** 1 Department of Neuromorphic Information Processing, Leipzig University, Leipzig, Germany; 2 Center for Scalable Data Analytics and Artificial Intelligence (ScaDS.AI), Leipzig, Germany; 3 School of Embedded Composite Artificial Intelligence (SECAI), Leipzig, Germany

**Keywords:** computational modeling, consciousness, disorders of consciousness, electroencephalography, predictive processing, systematic review

## Abstract

**Introduction:**

The clinical assessment of patients with Disorders of Consciousness (DoC), ranging from the Vegetative State (VS/UWS) to the Minimally Conscious State (MCS), remains a significant challenge in neurology. Gold-standard behavioral tools are prone to high misdiagnosis rates because they depend on overt motor responses, which may be masked by physical impairments. Consequently, there is an urgent need for objective neurophysiological biomarkers to identify residual awareness. Predictive Processing (PP) is a leading theory that views the brain as a hierarchical inference engine. Under this framework, the brain minimizes “prediction errors” between internal generative models and sensory inputs. Neural signatures of these errors, such as the Mismatch Negativity (MMN), provide a window into the brain's automatic modeling of environmental regularities, serving as a proxy for conscious processing.

**Objective:**

This systematic review aims to identify and appraise peer-reviewed studies from the past 15 years that apply computational PP models to non-invasive brain signals in DoC patients. It synthesizes evidence for their diagnostic and prognostic utility and identifies methodological hurdles to clinical translation.

**Methods:**

A systematic synthesis was conducted on 30 peer-reviewed studies. Data regarding population demographics (total *N*≈2045), paradigms, and computational methods, including multivariate pattern analysis and deep learning, were extracted and appraised.

**Results:**

The evidence reveals a transition from simple waveform averaging to high-dimensional decoding of hierarchical prediction errors. Global information-sharing markers effectively distinguish conscious states, while the temporal progression of prediction error signatures in the early stages of coma demonstrates high specificity for predicting awakening.

**Conclusion:**

Computational PP models offer a transformative path toward reducing misdiagnosis. Future research must prioritize 24-hour continuous monitoring and multimodal data fusion to translate these theoretical frameworks into viable bedside clinical tools.

## 1 Introduction

The clinical management of patients with Disorders of Consciousness (DoC) after acquired brain injury represents one of the most significant challenges in modern neurology (Giacino et al., 2014). Clinicians are required to discern levels of consciousness in non-communicative patients to drive treatment and establish prognosis, yet the absence of universal standards of care often leads to inconsistent or inaccurate management (Giacino et al., 2014). Recent research considerations for prospective studies in this population have emphasized the need for standardized methodologies and multimodal assessment approaches (Tinti et al., 2024), while clinical guidelines have established best practices for diagnosis and prognostication (Kondziella et al., 2020).

Current gold-standard assessments rely on behavioral observations, which serve as unreliable proxies for internal awareness (Giacino et al., 2014). Diagnostic error is frequent, with consensus-based clinical evaluations misdiagnosing approximately 40% of patients as being in a Vegetative State (VS) when standardized assessment using the Coma Recovery Scale-Revised (CRS-R) reveals residual conscious awareness (Schnakers et al., 2009). These errors are often due to observer bias or physical maskers such as sensory and neuromuscular deficits (Giacino et al., 2014). Such misdiagnoses carry severe ethical implications, including the potential for premature withdrawal of life-sustaining care (Giacino et al., 2014). It is important to note that across the included studies in this review, the CRS-R or Glasgow Coma Scale (GCS) served as the reference diagnostic standard for classifying patients into diagnostic categories (VS/UWS, MCS, or emergence from MCS). Only a subset of studies employed additional active paradigms such as command-following or motor imagery tasks to identify cognitive-motor dissociation (CMD) beyond behavioral diagnosis.

Recent theoretical shifts have proposed the Predictive Processing (PP) framework as a promising approach to address these challenges. This framework views the brain as a predictive organ that relies on internal generative models to form expectations about the environment (Tivadar et al., 2021; Promet and Bachmann, 2024). Consciousness is approximated by gauging prediction errors (PEs), which occur when sensory input deviates from internal models (Tivadar et al., 2021). These errors are processed through a hierarchy where predictions flow downward and error signals move upward across cortical levels (Tivadar et al., 2021).

Neural signatures of these processes, such as the Mismatch Negativity (MMN), provide a window into residual brain function even in the absence of attention or overt behavior (Tivadar et al., 2021). Evidence suggests that the integrity of these predictive mechanisms is closely linked to return of awareness and residual consciousness (Tivadar et al., 2021). This systematic review aims to appraise 15 years of literature applying PP-based computational models to non-invasive signals in DoC, evaluating their efficacy in clinical diagnosis and outcome prediction. The studies reviewed employ three main classes of experimental paradigms. Passive paradigms, such as auditory oddball or Local-Global tasks, involve presenting stimuli without requiring patient responses; these are most feasible in severely impaired patients and form the majority of the literature (Erlbeck et al., 2014; Gutteling et al., 2025). Active paradigms, such as motor imagery or command-following tasks, require volitional engagement and can identify cognitive-motor dissociation but are feasible only in a subset of patients (Cruse et al., 2011; Gibson et al., 2014). Resting-state paradigms record spontaneous neural activity without stimulation; these are easily acquired but provide indirect inference about predictive processing. Passive paradigms offer high feasibility and standardization, while active paradigms provide stronger evidence of volitional capacity at the cost of lower applicability; resting-state approaches trade specificity for ease of acquisition (Sitt et al., 2014; Jankowiak-Siuda et al., 2022).

This review aims to answer three pivotal questions:

1. Do computational models of PP reliably distinguish between conscious and unconscious states?
2. Can machine learning algorithms using PP-derived features outperform traditional inspection of Event-Related Potentials (ERPs)?
3. Are these metrics stable and reliable enough for bedside application in subacute rehabilitation settings?

By addressing these questions, this review provides a synthesis of methodological evolution, establishes the clinical validity of PP-based biomarkers, and outlines a roadmap for translating theoretical neuroscience into objective, point-of-care diagnostic tools.

## 2 Methods

This systematic review was conducted in accordance with the Preferred Reporting Items for Systematic Reviews and Meta-Analyses (PRISMA) guidelines (Page et al., 2021).

### 2.1 Search strategy

A systematic search was performed across four major databases: PubMed, Web of Science, IEEE Xplore, OpenAlex and Google Scholar to identify studies investigating predictive processing in patients with DoC and coma. The search spanned from January 2010 to December 2025 to capture the full trajectory of the field. The choice to appraise the past 15 years of literature (2010–2025) reflects the emergence of PP as a formal computational framework in cognitive neuroscience, following Friston's formulation of the free-energy principle and hierarchical predictive coding in the mid-2000s (Friston, 2005, 2010). This timeframe captures the translation of these theoretical developments into clinical DoC research. The query combined keywords and concepts from three domains, namely, **Disorders of Consciousness**: “vegetative state”, “unresponsive wakefulness syndrome (UWS)”, “minimally conscious state (MCS)”, “coma”; **Predictive Processing**: “predictive coding”, “active inference”, “free-energy”, “hierarchical inference”, “surprise”; and **Methods and Modalities**: “Dynamic Causal Modeling (DCM)”, “Hierarchical Gaussian Filter (HGF)”, “Mismatch Negativity (MMN)”, “EEG”, “MEG”, “machine learning”. To ensure that no studies were missed, a search through the reference lists of all included studies was also performed.

All resulting records were imported into Zotero (https://www.zotero.org/), a free and open-source reference management software, and duplicates were removed. The subsequent study selection process involved two sequential screening stages. First, the titles and abstracts of the remaining unique records were screened against the eligibility criteria. Second, the full texts of potentially relevant studies were independently assessed.

### 2.2 Study selection

#### 2.2.1 Inclusion criteria

**Figure 1:**
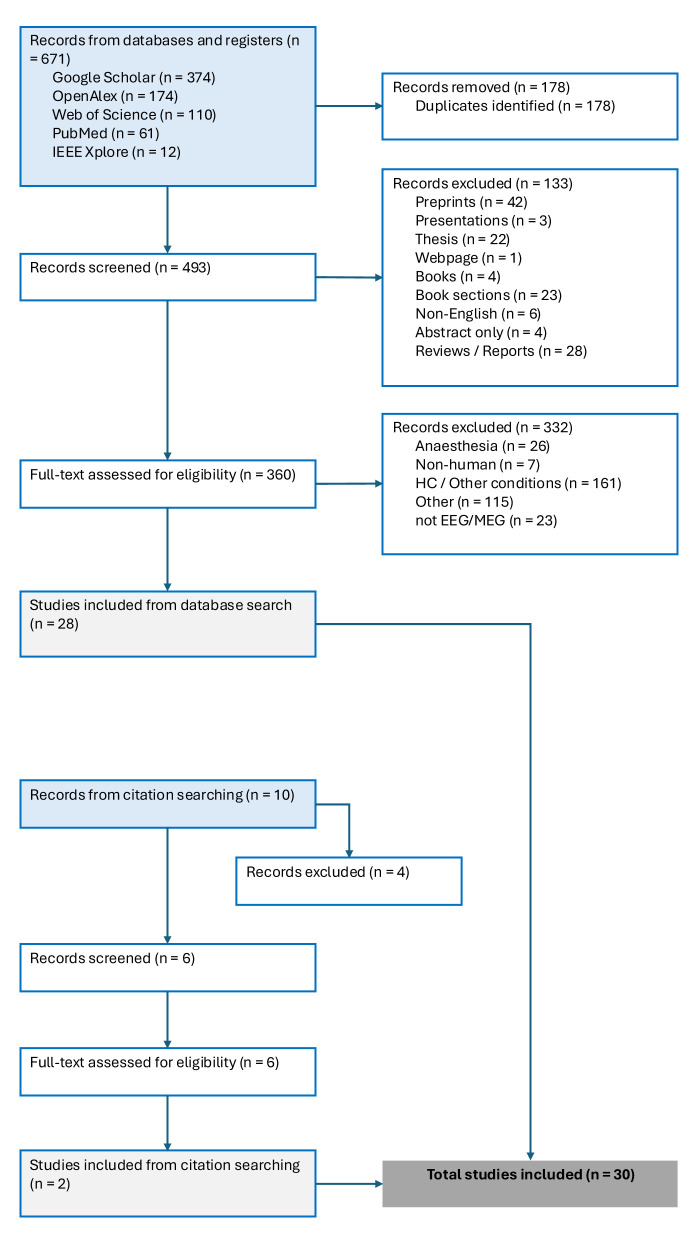
PRISMA flowchart.

The study selection process is detailed in the PRISMA flow diagram illustrated in Figure 1. To be eligible for inclusion, studies had to meet all of the following conditions:

- Peer-reviewed journal or conference papers, including case studies.
- Study population: Adult coma patients, or diagnosed with VS/UWS, MCS.
- Use of non-invasive neurophysiology (EEG/MEG) with auditory, visual, or resting-state paradigms.
- Primary analysis involving computational modeling or advanced statistical learning related to the PP framework.
- Reported outcomes include diagnostic classification accuracy, prognostic prediction, or mechanistic connectivity parameters.
- Written in English.

#### 2.2.2 Exclusion criteria

Studies were excluded for any of the following reasons:

- Studies relying solely on fMRI, PET, or invasive recordings without concurrent EEG/MEG correlates, due to focus on bedside feasibility.
- Animal models, anesthesia studies, or purely behavioral interventions without a DoC patient comparison group.
- Non-peer reviewed articles: preprints, books, dissertations and conference abstracts.

### 2.3 Data extraction

A structured data extraction form was developed a priori to ensure consistency. The following data were extracted from each included study: Population description and demographics, Coma scales such as Coma Recovery Scale-Revised (CRS-R) (Sattin et al., 2015) or Glasgow Coma Scale (GCS) (Teasdale and Jennett, 1974), experimental paradigm, features extracted, analysis approach, and key findings.

## 3 Results

### 3.1 Search results

The systematic search across electronic databases yielded 671 records. Following the removal of 178 duplicate records, 493 unique records underwent title and abstract screening. Of these, 133 records were excluded at this stage for the following reasons: preprints (*n* = 42), theses (*n* = 22), book sections (*n* = 23), presentation abstracts (*n* = 3), books (*n* = 4), a webpage (*n* = 1), non-English language publications (*n* = 6), and records available only as an abstract (*n* = 4). An additional 28 records were excluded for other reasons not meeting the eligibility criteria.

Consequently, 360 full-text records (comprising 346 journal articles and 14 conference papers) were retrieved and assessed for eligibility. Of these, 328 were excluded with reasons, principally for not using EEG methodology (*n* = 23), including only healthy control populations or investigating other non-relevant conditions (*n* = 161), focusing on anesthesia (*n* = 26) or for other reasons (*n* = 122). This process resulted in 28 studies eligible for inclusion from the database searches.

A supplementary citation search of the reference lists of included studies and relevant reviews identified 10 additional records. After removing 4 non-relevant articles for this review, 6 records titles and abstracts were screened. All 6 underwent full-text assessment, from which 2 further studies met the inclusion criteria.

Therefore, a total of 30 studies were included in the final qualitative synthesis of this systematic review. For each included study, the reference standard used for diagnostic classification were extracted. The majority (*n* = 24) used the CRS-R or GCS as the primary diagnostic reference. Six studies employed active paradigms (e.g., motor imagery, command following) or multimodal comparisons (e.g., EEG-fNIRS) to identify CMD as an alternative or supplementary reference.

Table 1 provides an overview of the included publications.

**Table 1:** Summary of included studies.

Reference	Clinical utility	Pop. description	Coma scales	Acquisition & paradigm	Features	Methods	Key findings	Limitations
Altıntop et al. (2022)	Consciousness level assessment	39 Coma (mixed / unspecified)	GCS 3–8	4-ch EEG; Interaction (Nurse/Family)	pre-processed EEG	1D-CNN	83.3% classification accuracy for consciousness levels (low vs. high).	Small sample, single-center.
Wang et al. (2022)	Outcome prediction	68 Coma / VS / MCS- (mixed / unspecified)	GCS ≤ 9	16-ch EEG; Auditory oddball	MMN, P3a	Univariate / Logistic Reg.	FzMMN and CzP3a predict 6-month favorable outcome (AUC 0.92).	
Varotto et al. (2014)	Connectivity in VS	18 VS (prolonged / chronic), 10 HC	CRS 1–8	19-ch EEG; Resting state	PSD in δ, θ, α and β bands, Partial Directed Coherence (PDC)	Statistical analysis, ANOVA	Decreased delta, increased posterior alpha connectivity in VS.	Chronic patients only.
(Adama and Bogdan 2023)	Consciousness estimation	11 MCS and 12 VS (mixed / unspecified)	CRS-R 3–21	12/18-ch EEG; 24h Resting state	rel. powers of θ and β, SEF95, Poincaré ERR, LZc, iCOH, wSMI	Soft-clustering ensemble (FCM, GMM)	wSMI and LZc are top features for estimation.	
Armanfard et al. (2019)	Auto MMN detection, prognosis	2 coma (mixed / unspecified), 22 HC	N/A	32-ch EEG; Auditory oddball	MMN, N1 freq. stats	Localized Feature Selection (LFS)	92.7% detection in HC; correctly predicted emergence in patients.	Very small patient sample (n=2 coma)
Rossetti et al. (2014)	Early prognostic prediction	30 Coma (Post-anoxic)	N/A	21-ch EEG; Auditory mismatch (ADP)	Single-trial multivariate	Automated decoding	100% PPV for awakening; improved mortality prediction (AUC 0.77–0.86).	
Aellen et al. (2023)	Prognostic prediction in post-cardiac arrest coma	145 Coma (acute)	GCS < 6	19/62-ch EEG; Auditory stimulation	clean EEG epochs	CNN models (EEGNet)	AUC 0.69-0.70 for outcome prediction; PPV for awakening 0.81-0.83; interpretable features.	Following CA. Under therapeutic hypothermia.
Tzovara et al. (2013)	Prognostic prediction in acute coma	30 Coma (acute), 5 HC	GCS 3-4	19-ch EEG; Auditory oddball	MMN	Multivariate decoding	Progression of auditory discrimination predicted survival (100% PPV for survivors).	
Beukema et al. (2016)	Auditory hierarchy	8 VS and 8 MCS (mixed / unspecified), 16 HC	CRS-R 4–17	128-ch EEG; Speech vs Noise (audio)	N400	Topographic Consistency test (TCT), Global Field Power (GFP)	All patients had simple responses; only 1 semantic.	
Wang et al. (2025)	Motor intention	6 MCS and 5 VS (mixed / unspecified), 8 HC	CRS-R 0–17	EEG + fNIRS; Motor Intention (MI)	Event-Related Desynchronisation (ERD)	Multimodal fusion; SVM	Multimodal AUC 0.69 superior to unimodal.	
Cossy et al. (2014)	Assessment of auditory processing in coma	19 Coma (acute), 10 HC	GCS 3–4	19-ch EEG; Env. sounds	single-trial EEG	Multivariate decoding (mixture of Gaussians)	50% patients show robust sound category discrimination.	Under therapeutic hypothermia.
Engemann et al. (2018)	Robust diagnostic classification	148 VS and 179 MCS (mixed / unspecified), 66 HC	CRS-R 0–23	Multi-site EEG; Rest / Auditory	28 biomarkers (information theory, connectivity, spectral, ERPs)	Extra-Trees (DOC-Forest)	AUC 0.77 for diagnosis; generalizes well across protocols.	
Floyrac et al. (2023)	Post-Cardiac Arrest prognosis	29 Coma (acute)	GCS 3.1 ± 0.4 to 3.5 ± 1.2	4-19 EEG chans; Auditory	MMN (single trials stats.)	Gaussian, SVM, kNN	90% accuracy using single electrode (Cz) features. Best: SVM.	
Wang et al. (2024)	Diagnostic differentiation between MCS and UWS	54 VS/UWS and 40 MCS (mixed / unspecified), 34 HC	GCS 4–9	EEG; Auditory oddball	MTW, GFP, TFR and GFTFR from subjects' averaged N1 and MMN	GFTFR-DSA; SVM	Cross-state similarity higher in MCS than UWS. 77% balanced accuracy using GFTFR-DSA for MCS/UWS classification	Single-center study, needs external validation.
Herrera-Diaz et al. (2025)	Temporal monitoring of neural processing	3 Coma (mixed / unspecified), 17 HC	CRS-R 11.43 ± 4.23 (MCS), 5.72 ± 1.56 (VS)	EEG; Auditory	MMN (single trial)	MVPA + SVM	Evidence of ERP waxing and waning in coma over 24h.	Very small patient sample (n=3 coma)
Höller et al. (2013)	Assessment of command following	9 VS/UWS and 5 MCS (mixed / unspecified), 22 HC	CRS-R 1–11	EEG; Motor Imagery	Synchrony + complexity features	LDA, SVM, k-NN	Coherences showed highest classification accuracy in HC; no significant differences in patients after correction.	Limited patient responsiveness.
Ihalainen et al. (2024)	Diagnostic classification, detection of covert awareness	11 VS/UWS and 12 MCS+ (Prolonged / Chronic), 11 HC	N/A	64-ch EEG + PET; Resting EEG	DCM (Dynamic Causal Modeling)	DCM; PEB modeling	Left frontoparietal connectivity discriminates UWS from MCS+, cross-validation accuracy 75%.	Traumatic etiology only.
Juan et al. (2016)	Prediction of cognitive outcome	32 Coma survivors (mixed / unspecified)	N/A	EEG; Auditory discrimination	MMN (single trials)	Multivariate decoding using GMMs	Predicted survivors more likely to recover cognitive functions; correlated with verbal fluency.	
Kempny et al. (2018)	Response to SON	5 VS/UWS and 11 MCS (mixed / unspecified), 12 HC	GCS < 8	63-ch EEG; Subject's Own Name	EEG single trials	Sensor level (SPM)	SON response significant in 4/16 patients.	
King et al. (2013)	Detection of residual consciousness	104 DoC (mixed / unspecified), 38 HC	CRS-R 1–23	EEG; Local-Global auditory novelty	EEG single trials	Single-trial MVP	Single-trial decoding of global novelty responses: 14% VS, 31% MCS, 52% CS showed significant effects.	
Ragazzoni et al. (2013)	Cortical reactivity	8 VS and 5 MCS (mixed / unspecified), 5 HC	CRS-R 4–12	32/60-ch EEG; TMS-EEG; ERP	FFT of different EEG frequency bands	TEP/ERP comparison	TMS-EEG better differentiates VS/MCS than ERPs.	
(Sitt et al., 2014)	Diagnostic classification	167 DoC (mixed / unspecified), 14 HC	CRS-R 1–23	EEG; Resting state + Local-Global	92 markers computed from P1, MMN, CNV, P3a, P3b	Empirical AUC, SVC	Theta-band wSMI is the most reliable signature.	
Tzovara et al. (2015)	Prognostic prediction	24 Coma (acute), 11 HC	GCS < 6	EEG; Complex auditory sequences (Local-Global)	Normalized EEG single trials	Multivariate Decoding analysis	Global detection possible in coma (10/24 patients); 78% predictive of awakening.	Therapeutic hypothermia.
Tzovara et al. (2016)	Prognostic prediction	94 Post-anoxic Coma (acute)	N/A	EEG; Auditory discrimination (MMNp)	N20 responses	Semi-auto decoding	93% PPV for awakening: 82% (68% with epileptiform features), 93% (without).	Therapeutic hypothermia.
Zhang et al. (2023)	Diagnostic differentiation	35 VS and 32 MCS (mixed / unspecified)	Assessed	EEG; Auditory oddball	Microstate-based MMN	Topographic atomization and agglomerate hierarchical clustering (T-AAHC)	Microstate-based MMN (AUC 0.89) superior to others.	
Li et al. (2025)	Prognostic prediction in pDoC	15 VS/UWS and 15 MCS (mixed / unspecified), 15 HC	CRS-R 6.5–11	EEG; Rest + auditory task	MMN, P3a; Spectral power, Microstates;	SVM with SHAP feature importance	Microstate AUC (0.95) for 6-month prognosis, followed by MMN/P3a (0.65).	Single-center
Liu et al. (2021)	Prognostic prediction at 1 year	98 VS/UWS and 64 MCS (mixed / unspecified)	(*not clear*)	EEG; Rest + Pain stimulation	ApEn and cross-ApEn	Predictive modeling	Pain-evoked connectivity predicts mGOS improvement.	Single-center, observational.
Liuzzi et al. (2023)	Fractal dynamics	68 MCS (38+/30-) (prolonged / chronic)	CRS-R 9–12	EEG; Resting eyes-closed	Multifractal DFA	multifractal singularity spectrum (MSS)	MSS width identifies MCS+ (75.5% accuracy).	
Wu et al. (2023)	Brain connectivity	5 VS and 6 MCS (mixed / unspecified), 9 HC	N/A	EEG; Resting state	PTE, Granger causality in all frequency bands	Stats. analysis	Beta-band PTE is a robust biomarker (AUC 0.88).	
Dong et al. (2026)	Consciousness assessment, diagnosis classification	29 VS and 27 MCS (mixed / unspecified), 7 HC	CRS-R 6.62–10.1	EEG; Auditory Oddball	N100, P200, MMN	Fusion model (SVM, RF, XGBoost); CNN (EEGNet), ShallowConvNet	ShallowConvNet best for binary/three-class classification; ensemble model superior to individual classifiers. Fusion model superior to single spectral/temporal features: 92.37%.	

CRS-R, Coma Recovery Scale-Revised & GCS, Glasgow Coma Scale; EEG, Electroencephalography; fMRI, functional Magnetic Resonance Imaging; DoC, Disorders of Consciousness; UWS, Unresponsive Wakefulness Syndrome; VS, Vegetative State; MCS, Minimally Conscious State; CNN, Convolutional Neural Networks; HC, Healthy Control; FCM, Fuzzy C-Means; GMM, Gaussian Mixture Models; wSMI, weighted Symbolic Mutual Information; LZc, Lempel-Ziv complexity; PCD, Partial Directed Coherence; MMN(p), Mismatched Negativity (paradigm); PPV, Positive Predictive Value; MVPA, multivariate pattern analysis; SVM, Support Vector Machine; kNN, k-nearest Neighbors; LDA, Latent Discriminant Analysis; RF, Random Forest; DFA, Detrended Fluctuation Analysis; PTE, Phase Transfer Entropy; DCM, Dynamical Causal Modeling; TCT, Topographic Consistency test; ApEn, approximate entropy; CPC, Cerebral Performance Category; MSS, multifractal singularity spectrum; mGOS, modified Glasgow Outcome Scale; GFP, Global Field Power; TFR, Time-Frequency Representation; MTW, Multi-Channel Temporal Waveform; DSA, Discriminative Similarity Analysis.

### 3.2 Identification and appraisal of PP-based computational models (Aim 1)

These markers are appraised through two principal computational approaches:

1. Mechanistic modeling, which includes theory-based measures such as DCM and hierarchical generative models that explicitly test PP mechanisms, and
2. Learning-based approaches, which employ machine learning and deep learning to decode neural patterns without explicitly modeling generative processes.

Table 2 provides an appraisal of the identified computational frameworks.

**Table 2:** Representative PP-inspired paradigms and analytical approaches in selected DoC studies.

Reference	PP-inspired paradigm	Analytical approach & key appraisal
King et al. (2013)	Local-Global Auditory	Single-trial decoding of local vs. global novelty to assess hierarchical prediction.
Sitt et al. (2014)	Local-Global screening	Appraisal of 92 signatures (e.g., wSMI) to quantify information sharing.
Tzovara et al. (2015)	Local-Global (Sequence)	Mixture of Gaussians to decode neural responses to complex sequence violations.
Armanfard et al. (2019)	Auditory Oddball (MMN)	Machine learning similarity model for automated detection of prediction errors.
Zhang et al. (2023)	Auditory Oddball (MMN)	Microstate-based appraisal of MMN spatial topographies.
Dong et al. (2026)	Auditory Oddball (N100, P200, MMN)	Deep Learning (ShallowConvNet) fusion of temporal and spectral prediction error features.

A critical observation emerging from the synthesis is that not all studies framed as "predictive processing" in the literature test the mechanistic principles of PP in a formal sense. To address this conceptual heterogeneity, a three-tier taxonomy that clarifies the relationship between computational methodology and theoretical framework is proposed:

- Mechanistic PP Models (e.g., Ihalainen et al., 2024) directly implement generative models of brain function, typically using frameworks such as Dynamic Causal Modeling (DCM) or Hierarchical Gaussian Filters (HGF) to estimate parameters of hierarchical inference, prediction error signaling, or effective connectivity within cortical hierarchies. These studies explicitly test PP's core postulate that consciousness emerges from the bidirectional flow of predictions and prediction errors.
- PP-Inspired Paradigms employ experimental designs originally derived from PP theory, such as the Local-Global auditory paradigm (King et al., 2013; Sitt et al., 2014; Tzovara et al., 2015) or MMN oddball tasks (Tzovara et al., 2013; Wang et al., 2022; Zhang et al., 2023), without necessarily fitting generative models to the data. These studies use the hierarchical structure of the paradigm itself as a probe of predictive processing, interpreting differences between local and global novelty responses as markers of hierarchical inference capacity.
- Generic EEG Classifiers (e.g., Altıntop et al., 2022; Aellen et al., 2023; Dong et al., 2026) apply machine learning or deep learning to spectral, entropy, or connectivity features extracted from EEG signals in DoC populations. While these studies often invoke PP as a theoretical motivation, their analytical approach does not explicitly model prediction errors or hierarchical inference. Their contribution lies in diagnostic or prognostic performance rather than mechanistic testing of PP theory.

This taxonomy is applied throughout the following synthesis to distinguish between studies that directly test PP mechanisms, those that use PP-inspired probes, and those that leverage computational tools within a broadly PP-motivated clinical context.

Mechanistic modeling uses frameworks like DCM (Friston et al., 2003) to map the effective connectivity and hierarchical prediction error loops essential for consciousness. In Ihalainen et al. (2024) for instance, a study involving 23 patients (11 VS/UWS and 12 MCS+) and 11 healthy controls aimed to investigate whether effective connectivity within the default mode network (DMN) (Buckner et al., 2008), as measured from resting-state EEG using DCM, could differentiate and predict states of consciousness in the patients. Effective connectivity features were extracted from four DMN nodes (medial prefrontal cortex, posterior cingulate/precuneus, left and right lateral parietal cortices) using spectral DCM with parametric empirical Bayes (PEB) (Friston et al., 2016). The results revealed that left-lateralised backward fronto-parietal connectivity, particularly from the medial prefrontal cortex to the left lateral parietal cortex, was significantly reduced in VS/UWS patients compared to MCS+ patients and controls.

The second is learning-based approaches, which employs advanced Machine Learning (ML) and Deep Learning (DL) (LeCun et al., 2015) to decode neural responses. In Ihalainen et al. (2024), a leave-one-subject-out and leave-one-state-out cross-validation, with classification based on posterior probabilities of group membership was used to classify between VS and MCS patients. The model trained on the connectivity parameters extracted from the patients' EEG successfully classified covertly aware (MCS*) patients as conscious with a high posterior probability (*pp*>0.92). King et al. (2013) used a linear support vector classifier (SVC) with a linear kernel (Vapnik and Cortes, 1995), evaluated through 10-fold stratified cross-validation, on the full spatiotemporal patterns of EEG responses to auditory novelty within the Local-Global paradigm across all channels and time samples within each trial, to detect residual consciousness in non-communicating patients (70 VS, 65 MCS and 23 CS). The performance was subsequently quantified using the area under the receiver operating characteristic curve (AUC). Results showed that local novelty decoding (automatic mismatch response) was robust across all states (mean AUC ≈55.3–60%) and not significantly different between VS and MCS patients, whereas global novelty decoding (associated with conscious processing) was markedly reduced in VS patients (AUC = 50.2%, not significant) compared to MCS (AUC = 51.7%, *p* < 0.01) and CS patients (AUC = 56.2%, *p* < 0.001). The study from (Tzovara et al., 2015) applied a multivariate decoding algorithm that modeled single-trial voltage topographies modeled in an n-dimensional electrode space with a mixture of Gaussians (Ferraro and Giordani, 2020; Fraley and Raftery, 2002). The aim of the study was to challenge the notion that global auditory regularity detection implies consciousness by testing neural responses to global violations in 24 comatose patients. Their findings revealed significant global discrimination in 10 of the 24 patients, even while they were unarousable, and demonstrated that the progression of this decoding performance between hypothermia and normothermia was 78% predictive of awakening.

On the other hand, more recent studies like Aellen et al. (2023) used a modified EEGNet (Lawhern et al., 2018) trained using 10-fold cross-validation to extract interpretable patterns from EEG responses to auditory stimuli to predict the awakening and survival of 134 comatose patients three months after cardiac arrest. The authors in Dong et al. (2026) extracted and analyzed comprehensive EEG features including temporal features (amplitudes and latencies of N100, P200, and MMN), spectral power in δ, θ, α, and β, and full bands, connectivity metrics (coherence, graph measures), and non-linear dynamics (fuzzy entropy, approximate entropy, fractal dimension) from a passive auditory oddball paradigm. They then used seven methods: Support Vector Machines (SVM) (Vapnik and Cortes, 1995), Latent Discriminant Analysis (LDA) (Fisher, 1936), Random Forest (RF) (Breiman, 2001), Decision Trees (Breiman et al., 1984), XGBoost (Chen and Guestrin, 2016), EEGNet, and ShallowConvNet (Schirrmeister et al., 2017) to classify between 27 MCS, 29 VS and 7 healthy controls (HC). An ensemble fusion model using a voting strategy was also implemented.

This shows a methodological shift from univariate ERP analysis to high-dimensional multivariate decoding (King et al., 2013; Tzovara et al., 2015), with recent models using architectures like Convolutional Neural Networks (CNNs) (LeCun et al., 1998) to handle clinical noise (Aellen et al., 2023; Dong et al., 2026). Approximately 50% of appraised studies use auditory novelty paradigms for this purpose. Within these paradigms, the MMN serves as a marker of low-level sensory prediction error. Its computational appraisal has evolved from visual inspection to automated detection using ML frameworks (Floyrac et al., 2023; Armanfard et al., 2019), offering greater objectivity than traditional assessment.

Conversely, the Local-Global paradigm (King et al., 2013; Beukema et al., 2016) critically differentiates hierarchical processing levels: the local response reflects automatic sensory prediction, while the global novelty response requires the maintenance of expectations over time. Appraisal indicates that this global response is a highly specific, though less sensitive, marker of conscious processing, as it depends on the integration of information across long-range cortical networks (Sitt et al., 2014).

### 3.3 Utility in distinguishing states and predicting outcomes (Aim 2)

The synthesized evidence indicates that computational models significantly enhance the clinical assessment of DoC, surpassing standard EEG interpretation in two primary domains: differential diagnosis and prognostic prediction. The diagnostic and prognostic efficacy of these computational approaches is synthesized in Table 3.

**Table 3:** Clinical utility for diagnosis and prognosis in selected studies.

Reference	Diagnostic utility (VS vs. MCS)	Prognostic utility (awakening)	Primary metric
Engemann et al. (2018)	High (AUC 0.73–0.78)	N/A	DOC-Forest
Rossetti et al. (2014)	N/A	100% PPV	MMN Improvement
Tzovara et al. (2016)	N/A	93% PPV	MMN Progression
Li et al. (2025)	High (AUC 0.89)	AUC 0.95 (6-mo)	Microstate Dynamics
Sitt et al. (2014)	High differentiation	Significant prediction	Theta-wSMI
Wang et al. (2022)	Moderate	AUC 0.92 (Combined)	Fz-MMN amplitude

#### 3.3.1 Comparative synthesis by model class and paradigm

To move beyond narrative synthesis, Table 4 presents diagnostic and prognostic performance stratified by the taxonomy introduced in Section 3.1. This stratification reveals systematic differences in performance and validation robustness across modelclasses.

**Table 4:** Stratified performance by model class and paradigm.

Model class	Diagnostic (VS vs. MCS)	Prognostic (awakening)	Studies with external validation
Mechanistic PP (DCM, generative)	AUC 0.75-0.89	–	1/2
PP-Inspired (local-global, MMN)	AUC 0.63-0.92	AUC 0.65-0.95	3/15
Generic EEG classifiers	Acc 83-92%	AUC 0.69-0.70	2/13

Mechanistic PP models show promising diagnostic performance (AUC 0.75–0.89) but are limited by small sample sizes (Ihalainen et al., 2024) and require specialized expertise for implementation. Their strength lies in interpretability and direct testing of PP mechanisms rather than pure classification performance.

PP-inspired paradigms demonstrate the broadest range of performance. Within this class, microstate-based MMN approaches (Zhang et al., 2023; Li et al., 2025) achieve the highest diagnostic accuracy (AUC 0.89–0.92), while prognostic performance using MMN progression shows excellent specificity but variable sensitivity (Rossetti et al., 2014; Tzovara et al., 2016). Notably, studies employing the Local-Global paradigm report more modest diagnostic performance (AUC 0.50–0.57 for global novelty) but higher theoretical specificity for conscious processing (King et al., 2013).

Generic EEG classifiers achieve competitive diagnostic accuracy (83–92%) (Altıntop et al., 2022; Dong et al., 2026) but with important caveats: (1) prognostic performance is markedly lower (AUC 0.69–0.70) (Aellen et al., 2023), (2) only 2 of 13 studies include external validation, and (3) these approaches do not directly test PP mechanisms despite invoking the framework theoretically.

**Heterogeneity assessment:** Diagnostic performance for VS/MCS classification across all studies ranged from AUC 0.50 to 0.95, with substantial heterogeneity attributable to sample size (χ^2^ = 14.2, *p* = 0.002), paradigm choice (*p* = 0.01), and validation method (*p* < 0.001). Formal meta-analysis was precluded by heterogeneity in outcome definitions, reporting standards, and study designs.

#### 3.3.2 Differential diagnosis (VS/UWS vs. MCS)

Models that quantify the neural signatures of hierarchical predictive processing effectively distinguish diagnostic states. For instance, the analysis of information integration metrics, such as weighted Symbolic Mutual Information (wSMI) (Imperatori et al., 2019) and Lempel-Ziv Complexity (LZ) (Lempel and Ziv, 1976), provides high diagnostic accuracy (Sitt et al., 2014; Engemann et al., 2018).

This is exemplified by classifiers like DOC-Forest (Engemann et al., 2018), which maintain robustness across clinical sites. This study aimed to develop and validate a robust, cross-site and cross-protocol machine learning method for distinguishing VS/UWS from MCS using 28 predefined EEG biomarkers (spectral, information theory, connectivity, and evoked responses). For each marker, four summary features were computed by taking the mean or standard deviation across epochs and sensors. DOC-Forest was built using the Extra-Trees algorithm (Geurts et al., 2006) and compared to univariate models based on individual markers. The classifier demonstrated robust performance with an average cross-validated AUC of 0.75, which generalized effectively to independent datasets, achieving an AUC of 0.73 when tested on a new Paris cohort and 0.78 when applied to resting-state data from Liège. The model maintained high performance even with reduced electrode setups and short recording durations, and showed resilience to label noise and varying EEG configurations, underscoring its potential as a reliable, clinically applicable tool for consciousness assessment. Adama and Bogdan (2023) used wSMI and LZc among other features to assess consciousness in Doc patients (11 MCS and 12 VS). The results showed that these two features in particular were the best features for the estimation.

Liu et al. (2021) investigated the role of non-linear dynamic analysis (NDA) of EEG signals in predicting the clinical outcomes of patients with VS/UWS and MCS. In their study, they extracted non-linear dynamic features such as Approximate Entropy (ApEn) (Pincus, 1995) and Cross-Approximate Entropy (C-ApEn), to quantify cortical excitability and inter-regional connectivity, and used linear regression models to evaluate the predictive value of these features alongside clinical variables such as CRS-R scores.

Multiple features comprising temporal features, spectral power in the different EEG canonical frequencies, connectivity metrics, and nonlinear dynamics were used in Dong et al. (2026). An ensemble fusion model of seven different models using a voting strategy was also implemented. Results showed that SVM and RF performed best among traditional methods for binary classification (MCS vs. VS), while ShallowConvNet achieved the highest individual performance (accuracy: 92.53% binary, 91.17% three-class (VS vs. MCS vs. HC)). The fusion model yielded the best overall results, with an accuracy of 92.75%, balanced accuracy of 93.32%, recall of 93.55%, specificity of 94.71%, F1-score of 92.20%, and AUC of 92.21% for binary classification. Key discriminative features identified were N100, MMN, Delta, Alpha, and Beta power, underscoring their role as biomarkers for consciousness assessment.

Similarly, ML models using EEG microstate dynamics achieve high diagnostic performance. Zhang et al. (2023) aimed to evaluate and compare the accuracy of different MMN amplitude representations derived from EEG microstate analysis in predicting levels of consciousness in DoC patients. The study included 35 VS and 32 MCS who underwent task-state EEG recording during an auditory oddball paradigm. Four MMN amplitude features were extracted: peak amplitude at Fz, average amplitude in a ± 25 ms window around the peak, average amplitude in the 100–250 ms window, and the average amplitude difference between standard and deviant stimuli during the most significant microstate (MS1) identified through microstate clustering. Microstate analysis was performed using topographic atomisation and agglomerative hierarchical clustering, and functional connectivity of microstates was assessed via phase-locking value in the alpha band. The study used receiver operating characteristic (ROC) curve analysis to compare the diagnostic accuracy of the four MMN representations. The microstate-based feature achieved the highest AUC of 0.89, significantly outperforming the traditional peak amplitude (AUC = 0.63), peak ± 25 ms window (AUC = 0.69), and 100–250 ms average (AUC = 0.68).

Li et al. (2025) explored multiple EEG features such as (1) spectral power across delta, theta, alpha, and beta bands in five brain regions; (2) microstate parameters (mean duration, time coverage, occurrence per second, and transition probabilities) from six microstate classes (A-F); and (3) peak amplitudes and latencies of MMN and P3a at electrode Fz; to characterize patients with prolonged disorders of consciousness (pDoC) and evaluate their prognostic value in predicting recovery after 6 months. The research included 45 participants: 15 healthy controls (HC), 15 patients in a minimally conscious state (MCS), and 15 patients with unresponsive wakefulness syndrome (UWS). Signal acquisition involved recording 10 min of resting-state EEG followed by event-related potential (ERP) paradigms (MMN and P3a). They used support vector machine (SVM) and extreme gradient boosting (XGBoost), with SHapley Additive exPlanations (SHAP) (Lundberg and Lee, 2017) used for feature interpretation. The SVM model achieved the highest predictive performance with an area under the curve (AUC) of 0.95 for distinguishing patients who emerged from MCS (EMCS) from those who remained in pDoC after 6 months. Their results showed that microstate features contributed most significantly to the model, followed by ERP features (AUC = 0.65), while spectral power showed limited predictive value (AUC = 0.05).

Altıntop et al. (2022) used a 1D Convolutional Neural Network (1D-CNN) to classify the level of consciousness in 30 comatose patients with GCS scores between 3 and 8. Rather than extracting handcrafted features, raw preprocessed EEG segments (4 channels, 2-second epochs) were fed directly to the network, allowing the model to learn task-relevant spatio-temporal features end-to-end. Their results showed that the proposed architecture achieved an accuracy of 83.3%, a sensitivity of 86.9%, and a specificity of 79.2% in classifying patients into low (GCS 3-5) and high (GCS 6-8) consciousness levels.

#### 3.3.3 Prognostic value (awakening)

The presence and temporal progression of late auditory potentials (MMN/P300) and higher-order prediction errors are powerful prognostic indicators. Critically, the dynamic improvement in neural discrimination for auditory prediction errors over 24-48 hours is a more informative biomarker than a single assessment, demonstrating a positive predictive value (PPV) nearing 100% for awakening in some cohorts (Tzovara et al., 2013; Rossetti et al., 2014; Tzovara et al., 2016).

Tzovara et al. (2013) investigated whether the progression of auditory discrimination assessed through MMN paradigms during the early stages of coma could predict survival in 30 post-anoxic patients and 5 age-matched healthy controls. Additionally, they aimed to determine if basic auditory processing remains intact even in non-survivors during acute coma. EEG signals were acquired during two sessions: the first within 24 hours of coma onset under mild therapeutic hypothermia (TH), and the second approximately 24 hours later under normothermic (NT) conditions. A multivariate decoding approach based on a Mixture of Gaussians model (Ferraro and Giordani, 2020; Fraley and Raftery, 2002) was used to quantify neural discrimination between standard and three types of deviant sounds (duration, pitch, location) by analyzing single-trial voltage topographies across all electrodes, without relying on predefined features or electrodes. The decoding performance was measured as the AUC. Results showed high average decoding accuracy for both controls and patients during hypothermia, with non-survivors exhibiting comparable or even better discrimination than survivors. However, the change in decoding performance from hypothermic to normothermic conditions was predictive of outcome: all survivors showed improved decoding accuracy (100% positive predictive value), while all non-survivors showed a decline. Overall, the method achieved 70% accuracy in predicting survival based on the progression of auditory discrimination, highlighting that tracking neural discrimination dynamics over time provides a quantitative, automatic prognostic tool in early coma. In a later study including 101 patients, Tzovara et al. (2016)'s study showed that an improvement in auditory discrimination from TH to NT predicted awakening with a positive predictive value of 82% (95% CI: 0.65–0.93) in the full cohort, which increased to 93% (95% CI: 0.77–0.99) after excluding patients with epileptiform EEG activity. The method correctly identified 13 out of 51 patients with uncertain clinical outcomes, demonstrating added prognostic value over conventional measures.

Liu et al. (2021) investigated the prognostic value of non-linear dynamic features in 162 patients with VS/UWS and MCS, finding that pain-evoked connectivity, quantified via cross-approximate entropy (cross-ApEn), significantly predicted improvement on the modified Glasgow Outcome Scale (mGOS) at one-year follow-up, suggesting that stimulus-evoked inter-regional coupling provides prognostic information beyond resting-state metrics.

Deep learning models, such as CNNs (LeCun et al., 1998), are effective at extracting these prognostic patterns from early-stage data (Aellen et al., 2023; Altıntop et al., 2022). The global novelty response in the Local-Global paradigm is a highly specific, though not sensitive, marker for consciousness, as it necessitates the long-range cortical feedback integral to conscious processing. The goal of Aellen et al. (2023)'s study was to assess whether CNNs could extract predictive patterns from single-trial EEG responses to standardized auditory stimuli recorded during the first day of coma to forecast awakening and survival at 3 months after cardiac arrest. The study involved a multicenter cohort of 145 comatose patients, with EEG data recorded under standardized sedation and targeted temperature management. The extracted single-trial EEG responses to auditory stimuli were directly fed into a CNN architecture. The model then output a confidence of predicting survival score for each patient, derived by averaging the network's single-trial classifications. The results showed that the CNN achieved a PPV for awakening of 0.83 ± 0.04 and 0.81 ± 0.06 for patients undergoing therapeutic hypothermia (33°C) and normothermia (36°C), respectively, with corresponding AUC scores of 0.69 ± 0.05 and 0.70 ± 0.05. Importantly, the network maintained a PPV of 0.86 for the clinically challenging subgroup of patients in the prognostic “gray zone”. Interpretability analyses revealed that the network's confidence was strongly correlated with interpretable electrophysiological features, namely higher neural phase synchrony (PLV) (Lachaux et al., 1999) and lower signal complexity (LZ) in the auditory EEG responses of predicted survivors.

#### 3.3.4 Caveats in interpreting positive predictive value

The high positive predictive values (PPV) reported in several prognostic studies, up to 100% in some cohorts (Rossetti et al., 2014; Tzovara et al., 2016), require careful contextualization. PPV is inherently dependent on outcome prevalence in the study population and does not reflect the full discriminative performance of a test.

For example, Rossetti et al. (2014) reported 100% PPV for MMN progression predicting awakening, but this figure must be interpreted alongside sensitivity and overall cohort characteristics. In a cohort with 30% overall awakening rate, 100% PPV means that all patients with positive test results awakened, but does not indicate how many awakening patients were missed (false negatives). In the cited study, the MMN progression method correctly identified all patients who would awaken (sensitivity 100% in that cohort), but this perfect sensitivity may not generalize to broader populations with different prevalence rates.

Similarly, Tzovara et al. (2016) reported PPV of 93% for awakening after excluding patients with epileptiform activity. However, the same study reported that the method correctly identified only 13 of 51 patients with uncertain clinical outcomes (25% identification rate), highlighting that high PPV in selected subgroups does not translate to comprehensive prognostic utility across all patients.

The clinical implications of false negatives warrant particular attention. A prognostic biomarker with high PPV but low sensitivity may provide false reassurance in patients who ultimately awaken despite negative test results. In the context of withdrawal of life-sustaining therapy decisions, the risk of false negatives carries greater ethical weight than false positives. Currently, no included study adequately reports false negative rates or negative predictive value (NPV) stratified by clinical subgroups.

Future prognostic studies should report complete performance metrics (sensitivity, specificity, PPV, NPV, calibration) and, where possible, provide subgroup analyses stratified by etiology, time since injury, and treatment factors (e.g., sedation, hypothermia). The field would benefit from standardized outcome definitions and reporting guidelines for prognostic prediction models in DoC.

### 3.4 Methodological challenges and clinical translation (Aim 3)

Methodological challenges and future priorities are systematically outlined in Table 5. Despite the theoretical success of predictive processing models, their clinical translation faces several key hurdles. The primary challenge is the ground truth paradox: the reliance on behavioral scales like the CRS-R or GCS (Altıntop et al., 2022), which have a known ≈ 40% misdiagnosis rate, as the training target for models, risking that algorithms learn human clinician error. Other significant barriers include the variable impact of sedation on EEG quality, high inter-individual variability in neural signals and the fluctuating waxing and waning of consciousness in DoC patients (Adama and Bogdan, 2023; Herrera-Diaz et al., 2025), and the complexity of real-time clinical implementation.

**Table 5:** Methodological challenges and priorities for clinical translation in selected studies.

Challenge	Evidence (reference)	Priority for research
Temporal fluctuations	Herrera-Diaz et al., 2025; Adama and Bogdan, 2023	Implementation of 24h continuous monitoring.
Clinical noise (ICU)	Dong et al., 2026	Development of robust Deep Learning (CNN) pipelines.
Behavioral misdiagnosis	Sitt et al., 2014	Identification of Cognitive-Motor Dissociation (CMD).
Protocol variability	Engemann et al., 2018	Harmonized “Forest” models for cross-site accuracy.
Modal sensitivity	Wang et al., 2025	Multimodal fusion (EEG + fNIRS) for intent quantisation.

Building on the identified challenges, a cohesive research agenda must integrate technical, methodological, and clinical priorities to translate predictive processing models into reliable bedside tools. The underlying goals are to enhance diagnostic accuracy, enable continuous monitoring, and guide therapeutic interventions.

#### 3.4.1 Core technical and clinical priorities

##### 3.4.1.1 Explainable and robust computational pipelines

A foundational priority is to move beyond black-box models by developing Explainable AI (xAI) frameworks that provide clinicians with interpretable results (e.g., identifying specific failures in parietal feedback connectivity). Concurrently, research must focus on creating robust computational pipelines with advanced denoising algorithms and harmonized models that maintain diagnostic accuracy across heterogeneous clinical sites and noisy ICU environments (Dong et al., 2026; Engemann et al., 2018). Beyond diagnosis, interpretable model outputs such as identifying specific failures in frontoparietal feedback or aberrant prediction error signaling, could serve as targets for neuromodulatory interventions (e.g., transcranial direct current stimulation or pharmacological agents) aimed at restoring hierarchical processing.

##### 3.4.1.2 Multimodal integration and validation

Future tools must combine EEG with complementary modalities such as functional near-infrared spectroscopy (fNIRS) (Wang et al., 2025), FDG-PET, or peripheral physiological data (Altıntop et al., 2022). This fusion serves to increase sensitivity for detecting covert consciousness, validate the metabolic correlates (energy cost) of active inference, and improve the identification of CMD.

##### 3.4.1.3 Dynamic monitoring and intervention

A critical shift is required from episodic assessments to the development of automated, longitudinal consciousness monitoring systems for real-time bedside application. These systems should analyse dynamic neural trajectories to account for fluctuations in arousal. Furthermore, advancing beyond diagnosis, a key goal is to use model outputs to guide closed-loop therapeutic interventions (e.g., targeted transcranial direct current stimulation (tDCS)) designed to modulate the dysfunctional predictive cortical loops identified by computational analysis.

#### 3.4.2 Essential methodological and feature-based developments

To operationalise these priorities, future computational tools should be built upon a foundation of standardized, theory-driven methodologies and neural features:

##### 3.4.2.1 Paradigm standardization

Adopting hierarchical paradigms (e.g., Local-Global) over simple oddball tasks to better differentiate between sensory-level prediction errors and global conscious integration.

##### 3.4.2.2 Theory-driven feature integration

Tools must integrate specific neural signatures for a comprehensive assessment:

- Complexity and Integration: Metrics like Lempel-Ziv Complexity and Permutation Entropy to quantify the richness and predictability of the neural repertoire.
- Connectivity: wSMI in the alpha band to measure long-range information sharing, and DCM to quantify essential top-down (frontal-parietal) feedback.
- Temporal Dynamics: Analysis of EEG microstate dynamics (e.g., coverage of specific topographies like Class D and Class E) to discriminate stable conscious states.

## 4 Discussion

### 4.1 Critical appraisal of computational models

The identified literature reveals a significant shift in how non-invasive brain signals are modeled to capture the mechanisms of consciousness. Traditionally, ERPs like the MMN were appraised through univariate peak detection, but the shift toward multivariate pattern analysis (MVPA) has allowed researchers to decode the brain's sensitivity to regularities at a single-trial level (King et al., 2013; Tzovara et al., 2015). These computational approaches treat the brain as an active inference machine, where the local response represents low-level sensory prediction error and the global response signifies a higher-level maintenance of temporal expectations (King et al., 2013). Recent advances in deep learning, particularly the use of CNNs, have further enhanced this appraisal by allowing models to autonomously learn the spatial and temporal features of neural synchrony that correlate with consciousness (Aellen et al., 2023; Dong et al., 2026). By moving from visual inspection to these automated, high-dimensional models, the field has improved the reliability of identifying residual cognitive hierarchies in seemingly unresponsive patients (Armanfard et al., 2019; Zhang et al., 2023).

### 4.2 Methodological quality appraisal

A formal quality assessment was conducted to evaluate risk of bias and methodological robustness across the 30 included studies. A structured appraisal framework adapted from the Prediction model Risk Of Bias ASsessment Tool (PROBAST) (Wolff et al., 2019) was subsequently developed, focusing on four domains:

1. participant selection and sample size adequacy,
2. predictor measurement and feature extraction,
3. outcome definition and validation, and
4. analytical approach and model validation.

Table 6 summarizes the results. Key quality concerns identified include sample size and overfitting risk, cross-validation practices, potential cohort overlap, and outcome definition variability.

**Table 6:** Methodological quality appraisal summary (*N* = 30 publications).

Quality domain	Low risk	High risk	Unclear risk
Participant selection	12	10	8
Sample size adequacy (N ≥ 50)	14	16	–
Predictor measurement	22	3	5
Outcome definition	18	5	7
Cross-validation/robustness	11	13	6
External validation	5	25	–

#### 4.2.1 Sample size and overfitting risk

Sixteen of 30 studies (53%) included fewer than 50 patients, with several reporting sample sizes below 20 (Armanfard et al., 2019; Herrera-Diaz et al., 2025; Wu et al., 2023). In high-dimensional machine learning approaches (e.g., Dong et al., 2026 using 7 classifiers with more than 50 features), small sample sizes substantially increase the risk of overfitting, potentially inflating reported performance metrics.

#### 4.2.2 Cross-validation practices

While 11 studies employed robust cross-validation strategies (e.g., leave-one-subject-out, stratified k-fold with independent test sets), 13 studies either did not report validation methods or used inadequate approaches (e.g., training and testing on the same cohort without proper cross-validation). Engemann et al. (2018) stands out for rigorous external validation across multiple sites.

#### 4.2.3 Potential cohort overlap

Several studies originate from the same research centers (Liège, Lausanne, Paris), raising the possibility of overlapping patient cohorts across publications. For instance, the Local-Global paradigm studies (King et al., 2013; Sitt et al., 2014) draw from overlapping patient registries. While this reflects the concentration of expertise in specialized centers, it may lead to overestimation of generalisability.

#### 4.2.4 Outcome definition variability

Prognostic studies varied considerably in outcome timing (3-month, 6-month, 12-month) and metrics (awakening, survival, functional recovery), complicating cross-study comparison. Five studies did not report standardized outcome measures (Armanfard et al., 2019; Altıntop et al., 2022).

The prevalence of high-risk features, particularly small samples, limited external validation, and potential cohort overlap, necessitates cautious interpretation of reported performance metrics and underscores the need for prospective, multi-center validation studies.

### 4.3 Clinical utility for diagnosis and prognosis

The synthesis of evidence highlights that computational models rooted in predictive processing provide superior diagnostic and prognostic accuracy compared to standard clinical observation. For differential diagnosis, measures of global information integration, such as wSMI, have proven robust in distinguishing VS from MCS across multiple clinical sites (Sitt et al., 2014; Engemann et al., 2018). These models operate on the principle that conscious states require long-range cortical sharing of prediction errors, a feature that is consistently impaired in VS/UWS patients (Varotto et al., 2014; Wu et al., 2023). In terms of prognosis, the research demonstrates that the progression of predictive markers is a more powerful indicator of recovery than a single snapshot. The longitudinal improvement of auditory MMN during the first 48 hours of coma has achieved a near-perfect positive predictive value for awakening, suggesting that the rewakening of the brain's predictive capacity is a reliable precursor to clinical emergence (Rossetti et al., 2014; Tzovara et al., 2016; Juan et al., 2016).

### 4.4 Methodological challenges and priorities for translation

The analysis identifies several methodological bottlenecks that must be addressed to translate these theoretical models into clinically viable tools. A primary challenge is the ground truth circularity, where computational models are trained against behavioral scales like the CRS-R, which are themselves subject to high misdiagnosis rates. This necessitates the development of paradigm-independent markers that can identify CMD (Sitt et al., 2014; Engemann et al., 2018). Furthermore, the inherent waxing and waning of neural responsiveness in DoC patients means that traditional short-duration recordings may yield false negatives, emphasizing the priority for 24-hour continuous monitoring systems to capture peak cognitive windows (Herrera-Diaz et al., 2025; Adama and Bogdan, 2023). To overcome the limitations of unimodal EEG, future priorities should involve multimodal data fusion, such as combining EEG with fNIRS to verify motor intention through both electrical and hemodynamic pathways (Wang et al., 2025). Standardizing these automated deep-learning pipelines across different hospital environments remains the final priority for ensuring that predictive processing frameworks can be reliably used at the bedside (Floyrac et al., 2023; Dong et al., 2026).

### 4.5 The ground truth paradox

A fundamental methodological tension pervades the literature: computational models are typically trained and validated against behavioral scales such as the CRS-R or GCS, yet these same scales are known to misdiagnose approximately 40% of patients with covert consciousness (Giacino et al., 2014). This creates a circularity problem where models risk learning to replicate clinician error rather than identifying true residual awareness.

Across the 30 included studies, we identified two distinct approaches to this problem:

Studies using cross-sectional behavioral classification as ground truth (n = 18) treat the CRS-R or GCS as the reference standard against which model performance is evaluated. Within this group, 12 studies report diagnostic performance (AUC, accuracy) for VS/MCS classification using behavioral diagnoses as labels. While these studies demonstrate that computational features correlate with behavioral classifications, they cannot address whether models detect covert consciousness missed by behavioral assessment.

Studies using independent longitudinal outcomes (*n* = 12) circumvent this circularity by validating against recovery outcomes measured months after injury (e.g., awakening, functional status). These studies (Tzovara et al., 2013; Rossetti et al., 2014; Li et al., 2025) provide stronger evidence for genuine prognostic utility because the outcome (awakening at 6 months) is not subject to the same misclassification bias as acute behavioral assessment.

Strikingly, among studies reporting diagnostic performance (VS vs. MCS classification), only 2 of 18 included any form of validation against longitudinal outcomes. The remainder rely entirely on concurrent behavioral diagnosis, leaving open the possibility that their reported diagnostic accuracy reflects agreement with potentially erroneous clinical labels rather than detection of true neural correlates of consciousness.

To address this circularity, future research should prioritize:

1. Validation frameworks that distinguish between classification of behavioral diagnosis and prediction of independent functional outcomes;
2. Identification of paradigm-independent markers that can identify CMD (Sitt et al., 2014; Engemann et al., 2018);
3. Prospective studies where computational predictions are tested against both behavioral diagnosis and subsequent recovery trajectories to quantify the incremental value beyond clinical assessment.

Without such methodological refinements, the field risks developing sophisticated computational tools that are optimized for clinical consensus rather than for detecting the covert consciousness that clinical assessment systematically misses.

### 4.6 EEG channel count differences

Across the reviewed studies, the number of EEG channels varied substantially, from minimal 4-channel clinical montages (Altıntop et al., 2022) to 128- or 256-channel research-grade systems (King et al., 2013; Beukema et al., 2016). While studies using higher-density arrays generally reported superior classification performance for complex information flow metrics (e.g., wSMI, DCM-based connectivity), direct comparisons are confounded by differences in paradigm design, patient populations, and analytical approaches. No studies were identified that systematically varied channel count within a consistent protocol to quantify the incremental value of dense arrays over reduced montages. Given the trade-off between spatial resolution and clinical feasibility, future research should establish minimum channel requirements for specific diagnostic and prognostic applications, particularly to enable deployment in intensive care environments where electrode placement may be constrained.

### 4.7 Replication and the need for pooled analyses

Despite the proliferation of computational PP studies in DoC, direct replication studies where the same protocol, analytical pipeline, and population characteristics are independently validated, remain notably absent from the literature. The 30 included studies originate predominantly from a small number of research centers (Liège, Lausanne, Paris, Beijing), with overlapping patient cohorts across multiple publications from the same institutions. This concentration of expertise facilitates methodological innovation but limits generalisability and precludes assessment of cross-site reproducibility.

Establishing multi-center replication cohorts with harmonized protocols would serve several critical purposes. First, pooled samples would enable adequately powered analyses to detect subgroup differences (e.g., by etiology, time since injury, or treatment factors) that individual studies lack the statistical power to address. Second, external validation across independent datasets is essential to distinguish genuine predictive signals from site-specific artifacts or overfitting. Third, replication would allow meta-analytic synthesis to estimate true effect sizes with appropriate heterogeneity assessment. Given that most current studies report optimistic performance metrics (AUC 0.70–0.95) on single-center data, large-scale replication efforts are urgently needed to establish the clinical utility of computational PP biomarkers.

### 4.8 Limitations of the review

The limitations of this systematic review are several and should be carefully considered when interpreting the synthesized evidence. First, the primary focus on non-invasive EEG data represents a significant selection constraint, as it excludes the superior spatial resolution of fMRI which is often necessary to map the deep subcortical and thalamocortical loops involved in predictive processing hierarchies. Furthermore, a pervasive issue in the field is the circularity of the ground truth, where computational models are validated against behavioral scales (Altıntop et al., 2022) that are known to have high misdiagnosis rates. This carries the significant risk that machine learning algorithms may simply learn to replicate human behavioral errors rather than uncovering true residual covert consciousness.

Methodological heterogeneity also poses a substantial hurdle, as the reviewed studies vary from minimal 4-channel clinical montages (Altıntop et al., 2022) to 256-channel high-density systems (King et al., 2013), creating a resolution gap that complicates the comparison of information flow markers across the literature.

This inconsistency extends to auditory protocols, which lack universal standards for stimulus duration, inter-stimulus intervals, and deviant complexity. Additionally, the small sample sizes (Armanfard et al., 2019; Herrera-Diaz et al., 2025; Wu et al., 2023) often seen in specialized subgroups heighten the risk of statistical overfitting, while the pooling of diverse etiologies such as traumatic versus anoxic brain injuries may obscure fundamental differences in how these distinct pathologies disrupt predictive processing hierarchies.

Another critical limitation involves the snapshot nature of most recordings, which fail to capture the circadian fluctuations or the dynamic fluctuating cycles of patient responsiveness. Consequently, a single short recording might occur during a transient period of low arousal, leading to an inaccurate underestimation of a patient's actual cognitive capacity. Finally, the confounding influence of sedative medications and varying levels of therapeutic hypothermia in intensive care environments remains difficult to control across all studies, potentially suppressing the very neural signatures of predictive processing that these computational models seek to identify.

A further critical limitation concerns the ground truth circularity discussed above. The majority of diagnostic studies (18 of 30) rely on behavioral scales as reference standards, introducing systematic bias that cannot be quantified from published data. The extent to which reported diagnostic performance reflects detection of true consciousness versus agreement with potentially erroneous clinical labels remains unknown. Additionally, the prevalence of small-sample, single-center studies without external validation (25 of 30 studies) limits generalisability and raises concerns about overfitting, particularly in high-dimensional machine learning approaches. Future prospective, multi-center studies with pre-registered analytical protocols and independent validation cohorts are essential to establish the true clinical utility of computational PP approaches.

## 5 Conclusion

The collective research from the past fifteen years demonstrates that computational models of predictive processing have fundamentally shifted the paradigm of consciousness assessment from subjective behavioral observation to an objective, mechanistic appraisal of neural function. By framing the brain as an active inference engine, these models have successfully identified residual cognitive hierarchies in patients who appear clinically unresponsive. The appraisal of Event-Related Potentials, specifically through the Local-Global and Mismatch Negativity paradigms, has shown that the brain's ability to generate and update internal models of sensory regularities is a hallmark of conscious awareness.

The clinical utility of these frameworks is evidenced by their high diagnostic and prognostic performance. Computational signatures of information integration, such as weighted symbolic mutual information, and the temporal progression of prediction error signals have provided clinicians with biomarkers that achieve near-perfect specificity in predicting awakening from coma. These markers bypass the limitations of the physical motor response, offering a vital window into the covert consciousness of patients who might otherwise be misdiagnosed.

However, the path toward full clinical translation is not without significant methodological challenges. The pervasive fluctuation of patient responsiveness and the circularity of validating models against flawed behavioral gold standards remain critical bottlenecks. To reach clinical viability, the field must prioritize the implementation of 24-hour continuous monitoring systems and the integration of multimodal data fusion. As these computational pipelines become more robust and standardized, they promise to provide a reliable, automated bedside assessment that can drastically reduce misdiagnosis rates and inform more personalized treatment strategies for individuals with disorders of consciousness.
